# HER3 PET Imaging: ^68^Ga-Labeled Affibody Molecules Provide Superior HER3 Contrast to ^89^Zr-Labeled Antibody and Antibody-Fragment-Based Tracers

**DOI:** 10.3390/cancers13194791

**Published:** 2021-09-24

**Authors:** Sara S. Rinne, Charles Dahlsson Leitao, Ayman Abouzayed, Anzhelika Vorobyeva, Vladimir Tolmachev, Stefan Ståhl, John Löfblom, Anna Orlova

**Affiliations:** 1 Department of Medicinal Chemistry, Uppsala University, 751 23 Uppsala, Sweden; sara.rinne@ilk.uu.se (S.S.R.); ayman.abouzayed@ilk.uu.se (A.A.); 2 Department of Protein Science, School of Engineering Sciences in Chemistry, Biotechnology and Health, KTH Royal Institute of Technology, 106 91 Stockholm, Sweden; chdl@kth.se (C.D.L.); ssta@kth.se (S.S.); lofblom@kth.se (J.L.); 3 Department of Immunology, Genetics and Pathology, Uppsala University, 751 85 Uppsala, Sweden; anzhelika.vorobyeva@igp.uu.se (A.V.); vladimir.tolmachev@igp.uu.se (V.T.); 4 Centrum for Oncotheranostics, National Research Tomsk Polytechnic University, 634050 Tomsk, Russia; 5 Science for Life Laboratory, Uppsala University, 751 23 Uppsala, Sweden

**Keywords:** HER3, PET, seribantumab, MM-121, zirconium-89, gallium-68, affibody molecules, monoclonal antibody, antibody-fragments, F(ab’)_2_

## Abstract

**Simple Summary:**

HER3 is a known driver for oncogenesis and therapy resistance in solid cancers. PET imaging could be a useful tool to non-invasively detect and monitor HER3 expression and aid in the selection of patients for HER3-targeted therapy. PET tracers based on therapeutic antibodies have thus far shown limited success in reliably imaging HER3-expressing tumors in clinical trials. Smaller-sized tracers specifically designed for imaging might be needed for higher contrast imaging and sufficient sensitivity. Our group has previously studied the use of radiolabeled affibody molecules for imaging of HER3 expression. In the present study, we compared four different types of potential PET tracers for imaging of HER3 expression in a preclinical model. We demonstrated that the affibody-based tracer, [^68^Ga]Ga-Z_HER3_, could provide overall superior imaging contrast to antibody- and antibody-fragment-based tracers shortly after injection. Our results indicate that HER3-targeting affibody molecules are promising agents for PET imaging of HER3 expression.

**Abstract:**

HER3 (human epidermal growth factor receptor type 3) is a challenging target for diagnostic radionuclide molecular imaging due to the relatively modest overexpression in tumors and substantial expression in healthy organs. In this study, we compared four HER3-targeting PET tracers based on different types of targeting molecules in a preclinical model: the ^89^Zr-labeled therapeutic antibody seribantumab, a seribantumab-derived F(ab)_2_-fragment labeled with ^89^Zr and ^68^Ga, and the ^68^Ga-labeled affibody molecule [^68^Ga]Ga-Z_HER3_. The novel conjugates were radiolabeled and characterized in vitro using HER3-expressing BxPC-3 and DU145 human cancer cells. Biodistribution was studied using Balb/c nu/nu mice bearing BxPC-3 xenografts. HER3-negative RAMOS xenografts were used to demonstrate binding specificity in vivo. Autoradiography was conducted on the excised tumors. nanoPET/CT imaging was performed. New conjugates specifically bound to HER3 in vitro and in vivo. [^68^Ga]Ga-DFO-seribantumab-F(ab’)_2_ was considered unsuitable for imaging due to the low stability and high uptake in normal organs. The highest tumor-to-non-tumor contrast with [^89^Zr]Zr-DFO-seribantumab and [^89^Zr]Zr-DFO-seribantumab-F(ab’)_2_ was achieved at 96 h and 48 h pi, respectively. Despite lower tumor uptake, [^68^Ga]Ga-Z_HER3_ provided the best imaging contrast due to the fastest clearance from blood and normal organs. The results of our study suggest that affibody-based tracers are more suitable for PET imaging of HER3 expression than antibody- and antibody-fragment-based tracers.

## 1. Introduction

The human epidermal growth factor receptor type 3 (HER3) is a target of increasing interest for molecular imaging in oncology. HER3 is known to be a marker for poor prognosis and a mediator of therapeutic resistance in several cancers, for example, breast and prostate cancer [1,2,3]. Currently, several clinical studies with HER3-targeting or co-targeting therapeutic agents are ongoing (clinincaltrials.gov (last accessed 10 August 2021), [3]). PET imaging of HER3 expression could be a valuable tool for improving the treatment of patients with HER3-expressing disease, allowing for non-invasive, repetitive whole-body detection and monitoring of HER3 expression as well as assessment of therapy response. However, development of high contrast agents for HER3 imaging is challenging due to the substantial HER3 expression in several healthy organs, e.g., salivary glands, organs of the GI tract, and particularly the liver (proteinatlas.org). Moreover, the level of overexpression of HER3 in tumors is relatively low, generally not exceeding 50,000 receptors/cell [4] (for reference, breast cancer patients are considered HER2+ with expression >2.3 × 10^6^ receptors/cell [5]).

Many factors need to be considered when developing potential molecular imaging agents. The choice of the targeting molecule (e.g., its size, polarity, and local charge) could appreciably influence the pharmacokinetics and biodistribution of the tracer and thereby the image contrast and sensitivity of the scan [6,7,8]. The ideal radiotracer should bind to the target receptor with high affinity, clear efficiently from the blood, and have minimal accumulation in non-targeted tissue [6,8]. Radiolabeled monoclonal antibodies were among the first tracers to be evaluated for molecular imaging of the HER-receptor family [8,9]. However, the slow extravasation and long residence time in blood are potential disadvantages for antibody-based imaging agents, because they result in an elevated background signal from blood-borne activity and increase the unspecific accumulation in non-targeted tissue [10]. Sufficient imaging contrast with antibody-based tracers is generally not achieved until several days after injection [11,12,13]. Decreasing the size of the imaging agent can provide better extravasation and faster clearance from the system, which may enable imaging with good contrast just a few hours after injection [6,14]. In recent years, a variety of smaller tracers based on different classes of targeting molecules have emerged as alternatives to antibody-based tracers and shown potential value and advantages as radionuclide-based agents for imaging of HER family members. These include peptides, antibody-fragments (e.g., Fab- and F(ab’)_2_-fragments and scFvs), single-domain antibodies (sdAbs, e.g., nanobodies), and engineered scaffold proteins (ESPs) [6,8,10].

Several of the HER3-targeting therapeutic antibodies under clinical and preclinical evaluation are being explored for PET imaging. [^89^Zr]Zr-lumretuzumab and [^89^Zr]Zr-GSK2849330 were able to visualize HER3 expression in patients 4–7 d pi [11,15]. [^89^Zr]Zr-lumretuzumab was able to detect 67.6% of HER3-expressing lesions with a size > 10 mm, but uptake quantification was only possible in 50% of the detected lesions [11]. [^89^Zr]Zr-GSK2849330 demonstrated dose-dependent tumor uptake in a small six-patient study [15]. With both of these ^89^Zr-labeled tracers, the image contrast was limited by the accumulation in normal tissue. Particularly the high uptake in the liver (due to the hepatobiliary excretion of antibodies) was considered problematic because it limits the detection of potential liver metastases. A clinical study with [^64^Cu]Cu-patritumab included 11 patients, but was terminated because of low uptake in HER3-expressing lesions [16]. More HER3-targeting antibodies for imaging have been studied in preclinical settings [17,18]. A few alternative HER3-imaging agents based on other targeting molecules have also been reported in preclinical studies. [^64^Cu]Cu-DOTA-mAb105-F(ab′)_2_ was able to detect changes in HER3 expression in response to therapy with AKT and PI3K inhibitors [19]. A biparatopic nanobody conjugate, [^89^Zr]Zr-MSB0010853, showed maximum tumor uptake in xenografts 24 h pi and imaged HER3 expression up to 96 h pi. The undecapeptide [^68^Ga]Ga-HER3P1 could visualize HER3 expression, but requires further optimization to increase the uptake in tumors [20].

More extensive preclinical work has been performed on the development of HER3-targeting affibody molecules, a class of ESPs. The small size (7 kDa) of affibody molecules presents a distinct advantage in the context of radionuclide-based imaging due to the more rapid blood clearance by renal filtration compared with antibody-based radiotracers. Additional benefits include high thermal and chemical stability and straightforward bacterial production [21]. Affibody molecules have been engineered to bind with high affinity and specificity to a multitude of targets including HER3. The affibody Z_HER3:08698_ has been developed to bind HER3 with low picomolar affinity and with cross-reactivity to murine ErbB3 [22]. Radiolabeled HER3-binding affibody molecules for PET and SPECT imaging have been used to visualize HER3 expression as early as 1 h pi, differentiate between high- and low-expressing xenografts models, and to detect changes in HER3 expression and receptor occupancy during HER3-targeted therapy [23,24,25,26]. Additional optimization of the molecular format and labeling approach resulted in major improvement in imaging contrast, particularly the tumor-to-liver contrast [27,28,29]. Among the studied variants, [^68^Ga]Ga-(HE)_3_-Z_HER3_-NODAGA and radiocobalt-labeled (HE)_3_-Z_HER3_-DOTA have shown the most promising characteristics for affibody-based imaging of HER3 expression thus far [27,30].

There might be perceived advantages in using radiolabeled versions of pre-existing therapeutic antibodies because, in addition to being used for target detection, they can also be used for dosing studies and imaging of receptor occupancy. However, for a difficult target such as HER3, smaller imaging agents, specifically designed for imaging, may provide a lower background signal, resulting in better contrast, enabling same-day scanning. Thus far, no side-by-side comparisons of different HER3-targeting diagnostic agents have been reported. The aim of this study was to compare the PET imaging properties of HER3-targeting tracers, based on three different molecules, at their respective favorable imaging time points for visualization of HER3 expression in a preclinical mouse model: a ^89^Zr-labeled version of the HER3-targeting therapeutic antibody seribantumab (MM-121), which, to our knowledge, has not yet been reported; a seribantumab-derived ^68^Ga and ^89^Zr labeled F(ab’)_2_-fragement, and the ^68^Ga-labeled affibody molecule [^68^Ga]Ga-Z_HER3_. This study demonstrated that, even at their respective favorable imaging time points, the achieved image contrast varied appreciably depending on the type of targeting molecule, due to the different pharmacokinetics and interactions with healthy tissue. Due the fast clearance and low accumulation in the background, the affibody-based tracer provided the overall best imaging contrast already 3 h pi.

## 2. Materials and Methods

### 2.1. General

HER3-expressing human cancer cell lines BxPC-3 (pancreatic cancer) and DU145 (prostate cancer) were obtained from ATCC (via LGC Promochem, Borås, Sweden). Cells were maintained in RPMI 1640 media (with L-glutamine, Biowest, Riverside, MO, USA) supplemented with 10% fetal bovine serum (Sigma–Aldrich, St. Louis, MO, USA) and 1% penicillin–streptomycin (Biochrom, Berlin, Germany).

^89^Zr (solution in 0.1 M oxalic acid) was purchased from Perkin Elmer (Waltham, MA, USA). ^68^Ga was obtained by elution of a ^68^Ge/Ga-generator (Cyclotron Co., Obninsk, Russia) with 0.1 M metal-free HCl. The radioactivity content in the samples was measured in an automated gamma counter with an NaI(TI) detector (1480 Wizard, Wallac, Finland). The statistical significance (*p* < 0.05) for in vitro and in vivo specificity experiments was assessed by a two-sided, unpaired *t*-test. One-way ANOVA with post-hoc *t*-test corrected for multiple comparisons using Tukey correction was used for statistical analysis of the biodistribution experiments.

All in vivo experiments were approved by the local authorities for laboratory animal welfare (Uppsala, approval number C4/16).

### 2.2. Generation of Seribantumab-F(ab’)_2_ and DFO Conjugation

Seribantumab was purchased from Evitria (Zurich, Switzerland). Seribantumab-F(ab’)_2_ was generated from enzymatic digestion using the Pierce™ F(ab’)_2_ Preparation Kit (Thermo Scientific, Chicago, IL, USA) according to the manufacturer’s instructions. Briefly, seribantumab (4.1 mg/mL) was incubated in a spin column containing immobilized pepsin equilibrated with digestion buffer at 37 °C for 6.5 h in a rotamixer. The antibody digest and immobilized pepsin were separated by centrifugation. Non-digested seribantumab and Fc fragments were separated from seribantumab-F(ab’)_2_ with size-exclusion chromatography (SEC) on a 1200 series HPLC system (Agilent Technologies, Santa Clara, CA, USA) using a Superdex^®^ 200 Increase 10/300 GL column (Cytiva, Uppsala, Sweden) and a flow rate of 0.5 mL/min of PBS. Successful removal of non-digested seribantumab and Fc fragments was evaluated with SDS-PAGE.

### 2.3. Conjugation of DFO to Seribantumab and Seribantumab-F(ab’)_2_

The buffer was changed to 0.1 M NaHCO_3_ (pH 9) for both seribantumab and purified seribantumab-F(ab’)_2_ using an Amicon^®^ Ultra-15 Centrifugal Filter Unit (Merck Millipore, Burlington, MA, USA). Conjugation to p-SCN-Bn-Deferoxamine (DFO, Macrocyclics, Houston, TX, USA) was performed at a molar excess of 4.4 and 2.2 for seribantumab and seribantumab-F(ab’)_2_, respectively, by overnight incubation at room temperature. The buffer was changed to 0.25 M NaAc (pH 5.5) using PD-10 desalting columns (Cytiva, Uppsala, Sweden).

### 2.4. Kinetic Evaluation of Seribantumab and Seribantumab-F(ab’)_2_

Binding kinetics of seribantumab and seribantumab-F(ab’)_2_ were evaluated with surface plasmon resonance (SPR) on a Biacore 8k system (Cytiva, Uppsala, Sweden) using a CM5 sensor chip immobilized with human HER3 (Sino Biological, Wayne, PA, USA) and murine ErbB3 (Sino Biological, Wayne, PA, USA). The experiment was performed with multi-cycle injections using five concentrations (100, 50, 25, 12.5, and 6.25 nM) at 25 °C and duplicate surfaces. Sensorgrams were analyzed with a Langmuir 1:1 kinetic model.

### 2.5. Radiolabeling of DFO-Seribantumab and DFO-Seribantumab-F(ab’)_2_ with ^89^Zr and ^68^Ga

DFO-seribantumab and DFO-seribantumab-F(ab’)_2_ were labeled with ^89^Zr using a modified version of the protocol previously published by Vosjan et al. [31]. In brief, ^89^Zr (0.036–0.048 MBq/µg for labeling of DFO-seribantumab, 0.072–0.08 MBq/µg for DFO-seribantumab-F(ab’)_2_ was mixed with 2 M Na_2_CO_3_ and incubated for 3 min at room temperature (with a volume ratio of 2:1 between ^89^Zr and Na_2_CO_3_), followed by the addition of 0.5 M HEPES. Thereafter, either 100 µg/0.7 nmol (42 µL) of DFO-seribantumab or 50 µg/0.45 nmol (42 µL) DFO-seribantumab-F(ab’)_2_ was added to the reaction mixture followed by an equal volume of 0.5 M HEPES. Reaction mixtures were then incubated for 1 h at 25 °C under gentle shaking. Radiochemical yield was determined by radio-iTLC (using silica-gel-impregnated glass micro-fiber chromatography paper, Agilent Technologies, Santa Clara, CA, USA) using 20 mM citric acid as the eluent. The activity distribution was measured using a Cyclone storage phosphor system (Packard) and analyzed by OptiQuant image analysis software (Perkin Elmer, Waltham, MA, USA). If necessary, radioconjugates were purified with size exclusion chromatography using Illustra NAP5 columns (GE Healthcare, Chicago, IL, USA). NAP-5 columns were pre-equilibrated with 1% BSA/PBS and eluted with PBS.

For labeling of DFO-seribantumab-F(ab’)_2_ with ^68^Ga, 50 µg of DFO-seribantumab-F(ab’)_2_ were added to 100 µL of 7.6 M sodium acetate buffer pH 4 and incubated with 40 µL ^68^Ga-eluate (20–40 MBq) for 10 min at 25 °C. Radiochemical yield was determined by iTLC, and purification was performed as described above.

### 2.6. Stability of [^89^Zr]Zr-DFO-Seribantumab, [^89^Zr]Zr-DFO-Seribantumab-F(ab’)_2_, and [^68^Ga]Ga-DFO-Seribantumab-F(ab’)_2_

To test the stability of the radiolabeled conjugates, a sample of the radiolabeling mixture (2 µg of [^89^Zr]Zr-DFO-seribantumab (96 kBq) and 1 µg of [^68^Ga]Ga/[^89^Zr]Zr-DFO-seribantumab-F(ab’)_2_ (57 kBq/160 kBq) were taken and incubated in excess PBS at room temperature or human serum at 37 °C. Samples were taken after 1 h, 3 h, and 24 h incubation and analyzed by iTLC.

### 2.7. Production and Radiolabeling of [^68^Ga]Ga-Z_HER3_

[^68^Ga]Ga-(HE)_3_-Z_HER3:08698_-NODAGA (hereafter referred to as [^68^Ga]Ga-Z_HER3_) was produced and labeled with gallium-68 according to previously published methods [27]. Radiochemical yield was determined by radio-iTLC. [^68^Ga]Ga-Z_HER3_ was used without additional purification steps.

### 2.8. In Vitro Characterization of [^89^Zr]Zr-DFO-Seribantumab, [^89^Zr]Zr-DFO-Seribantumab-F(ab’)_2_, and [^68^Ga]Ga-DFO-Seribantumab-F(ab’)_2_

HER3-expressing human cancer cells BxPC-3 and DU145 cells were used for the in vitro characterization of [^89^Zr]Zr-DFO-seribantumab and [^68^Ga]Ga/[^89^Zr]Zr-DFO-seribantumab. Cells were seeded in 35 mm cell dishes two days before the experiments. Experiments were performed in triplicates (*n* = 3).

Evaluation of in vitro specificity was performed according to previously published protocols [27]. Cells were incubated with either 0.5 nM of [^89^Zr]Zr-DFO-seribantumab, 0.5 nM [^89^Zr]Zr-DFO-seribantumab-F(ab’)_2_, or 0.1 nM of [^68^Ga]Ga-DFO-seribantumab-F(ab’)_2_ for 1 h at 37 °C. HER3 receptors were pre-blocked using 300–500-fold molar excess of seribantumab, seribantumab-F(ab’)_2_, or Z_HER3_ affibody. VEGFR-targeting antibody bevacizumab was added as a non-HER3-binding control. Uptake and internalization were studied by incubating cells with 0.5 nM of [^89^Zr]Zr-DFO-seribantumab, 0.5 nM [^89^Zr]Zr-DFO-seribantumab-F(ab’)_2_, or 0.1 nM of [^68^Ga]Ga-DFO-seribantumab-F(ab’)_2_, and at selected time points the membrane-bound and internalized activity were collected using the acid wash method as described earlier [29,32].

### 2.9. Biodistribution and In Vivo Specificity of [^68^Ga]Ga-DFO-Seribantumab-F(ab’)_2_, [^89^Zr]Zr-DFO-Seribantumab-F(ab’)_2_, and [^89^Zr]Zr-DFO-Seribantumab

Female Balb/c nu/nu mice were inoculated with BxPC-3 (HER3+, 7 × 10^6^ cells/mouse) or RAMOS (HER3–, 7 × 10^6^ cells/mouse) xenografts 24 days and 19 days before the experiment, respectively. At the time of the experiment, the average weight of BxPC-3 xenografts was 0.08 ± 0.06 g, and the average weight of RAMOS xenografts was 0.8 ± 0.4 g.

For biodistribution studies, mice bearing BxPC-3 xenografts were intravenously injected with equimolar protein doses corresponding to 27 µg for [^68^Ga]Ga/[^89^Zr]Zr-DFO-seribantumab-F(ab’)_2_ (0.04–0.05 MBq for ^89^Zr, 0.25 MBq for ^68^Ga), 35 µg [^89^Zr]Zr-DFO-seribantumab (0.05–0.08 MBq), and 2 µg [^68^Ga]Ga-Z_HER3_ (0.4 MBq). Non-labeled conjugate was used to adjust the protein dose to the desired amount. Time points for biodistribution were 3 h for [^68^Ga]Ga-DFO-seribantumab-F(ab’)_2_; 3 h, 24 h, and 48 h for [^89^Zr]Zr-DFO-seribantumab-F(ab’)_2_; 48 h and 96 h for [^89^Zr]Zr-DFO-seribantumab. A dual isotope approach was used to study the biodistribution of [^68^Ga]Ga/[^89^Zr]Zr-DFO-seribantumab-F(ab’)_2_ 3 h pi. At each time point, a group of *n* = 3–4 mice was sacrificed by heart puncture after intraperitoneal injection of a mixture of ketamine (250 mg/kg) and xylazine (25 mg/kg). Samples of blood, salivary glands, lung, liver, stomach, spleen, small intestine, kidneys, tumor, muscle and bone were collected, weighed, and measured for radioactivity content.

Mice bearing both HER3 positive BxPC-3 and HER3 negative RAMOS xenografts were used for the in vivo specificity test 3 h pi ([^68^Ga]Ga/[^89^Zr]Zr-DFO-seribantumab-F(ab’)_2_) and 96 h pi ([^89^Zr]Zr-DFO-seribantumab) and treated according to the biodistribution protocol described above.

### 2.10. Ex Vivo Autoradiography and Hematoxylin and Eosin Staining of BxPC-3 Xenografts

At the respective time points for biodistribution, BxPC-3 xenografts of mice injected with [^89^Zr]Zr-DFO-seribantumab-F(ab’)_2_, [^89^Zr]Zr-DFO-seribantumab, and [^68^Ga]Ga-Z_HER3_ were excised, halved, and frozen at −80 °C. Sectioning (20 µm thickness) and autoradiography were performed as described previously [33].

Hematoxylin and eosin staining of the tumor sections was performed following a standard protocol. After staining, the sections were scanned using PathScan Enabler IV (Meyer Instruments, Houston, TX, USA).

### 2.11. nanoPET/CT Imaging

BxPC-3 xenograft-bearing mice were intravenously injected with [^89^Zr]Zr-DFO-seribantumab-F(ab’)_2_ (1.18 MBq, 27 µg), [^89^Zr]Zr-DFO-seribantumab (1.38 MBq, 35 µg), and [^68^Ga]Ga-Z_HER3_ (7.05 MBq, 2 µg). Time points for PET-scans were matched with the time points of the biodistribution experiments. Mice were kept under general anesthesia (0.06% sevoflurane; 50%/50% medical oxygen:air) for scans at early time points and euthanized before the final scan.

Whole-body nanoPET images were acquired using a nanoScan PET/MR (Mediso Medical Imaging Systems Ltd., Budapest, Hungary). Scan times were 45–60 min for [^89^Zr]Zr-DFO-seribantumab-F(ab’)_2_ and [^89^Zr]Zr-DFO-seribantumab, and 30 min for [^68^Ga]Ga-Z_HER3_. A CT scan was performed immediately after the PET scan, using a nanoScan SPECT/CT (Mediso Medical Imaging Systems Ltd., Budapest, Hungary) with the same bed. The parameters for the CT scans were a 5 min acquisition time, an X-ray energy peak of 50 keV/670 μA, and 480 projections. Reconstruction of the PET scans was conducted using the Tera-Tomo™ 3D reconstruction engine with decay correction at the injection administration time. CT data were reconstructed using filter back projection in Nucline 2.03 Software (Mediso Medical Imaging Systems Ltd., Budapest, Hungary). PET and CT scans were fused using InterView FUSION software (Mediso Medical Imaging Systems Ltd., Budapest, Hungary).

## 3. Results

### 3.1. Generation of Seribantumab-F(ab’)_2_ and DFO Conjugation

Close to complete digestion was achieved for seribantumab following incubation with pepsin (Figure S1, third lane). Residual non-digested seribantumab and Fc fragments were successfully removed by SEC purification, shown by SDS-PAGE (Figure S1, fourth lane).

### 3.2. Kinetic Evaluation of Seribantumab-F(ab’)_2_ and Seribantumab

Seribantumab-F(ab’)_2_ retained its binding to both human HER3 and murine ErbB3 after pepsin digestion, shown by representative sensorgrams with fitted curves (Figure S2A,B, respectively). Average K_D_-values based on two surfaces with different immobilization levels are displayed in the sensorgrams. Seribantumab-F(ab’)_2_ and seribantumab demonstrated affinities in the low nanomolar range for both human HER3 and murine ErbB3 (mErbB3). Seribantumab-F(ab’)_2_ exhibited slightly higher affinity compared to seribantumab.

### 3.3. Radiolabeling and Stability

Labeling of all conjugates was successful. Radiochemical yields determined by radio-iTLC are shown in Table 1. [^89^Zr]Zr-DFO-seribantumab was used without further purification. Purity of [^89^Zr]Zr-seribantumab-DFO-F(ab’)_2_ after purification with NAP5-size exclusion columns was 97 ± 1%. The radiochemical purity of [^68^Ga]Ga-Z_HER3_ was 97 ± 2%.

No release of ^89^Zr was observed during incubation of [^89^Zr]Zr-DFO-seribantumab in PBS. Minor release of ^89^Zr from [^89^Zr]Zr-DFO-seribantumab-F(ab’)_2_ was observed after 24 h incubation in PBS. Both ^89^Zr-labeled conjugates showed almost a 25% release of protein-bound activity after 24 h in human serum. [^68^Ga]Ga-DFO-seribantumab-F(ab’)_2_ was stable when incubated in PBS, but after incubation in human serum for 3 h, the majority of the activity had dissociated from the protein.

### 3.4. In Vitro Characterization of [^89^Zr]Zr-DFO-Seribantumab, [^89^Zr]Zr-DFO-Seribantumab-F(ab’)_2_, and [^68^Ga]Ga-DFO-Seribantumab-F(ab’)_2_

**Figure 1:**
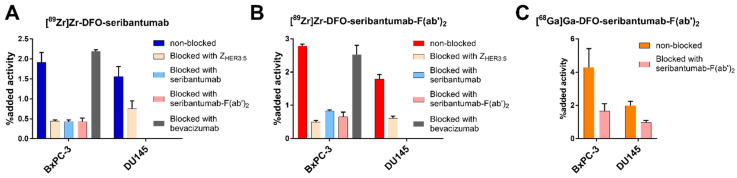
In vitro specificity of (**A**) [^89^Zr]Zr-DFO-seribantumab, (**B**) [^89^Zr]Zr-DFO-seribantumab-F(ab’)_2_, and (**C**) [^68^Ga]Ga-DFO-seribantumab-F(ab’)_2_ on BxPC-3 and DU145 cells. A blocking agent was added 15 min prior to the addition of the radiolabeled conjugates. The VEGFR-binding antibody bevacizumab was included as a non-HER3-blocking control. The in vitro specificity of [^68^Ga]Ga-Z_HER3_ was published earlier and can be found in reference [27].

All compounds demonstrated HER3-specific binding when tested on BxPC-3 and DU145 cells in vitro. Pre-saturation of HER3 receptors resulted in significantly lower uptake of [^89^Zr]Zr-DFO-seribantumab, [^89^Zr]Zr-DFO-seribantumab-F(ab’)_2_, and [^68^Ga]Ga-DFO-seribantumab-F(ab’)_2_ compared with the non-blocked control group in both BxPC-3 and DU145 cells (Figure 1). Taking into account the limitations associated with the short half-life of ^68^Ga and due to the confirmed cross-blockability shown in Figure 1B, we did consider blocking with Z_HER3_ to be sufficient to demonstrate HER3 specific binding for [^68^Ga]Ga in Figure 1C. Addition of the control antibody, VEGFR-targeting bevacizumab, did, as expected, not influence the uptake of [^89^Zr]Zr-DFO-seribantumab and [^89^Zr]Zr-DFO-seribantumab-F(ab’)_2_. In vitro specificity of [^68^Ga]Ga-Z_HER3_ was earlier reported [27].

**Figure 2:**
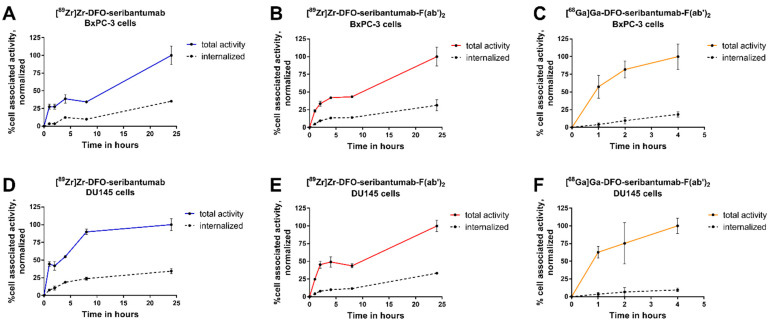
Cellular uptake and internalization of [^89^Zr]Zr-DFO-seribantumab (**A**,**D**), [^89^Zr]Zr-DFO-seribantumab-F(ab’)_2_ (**B**,**E**), and [^68^Ga]Ga-DFO-seribantumab-F(ab’)_2_ (**C**,**F**) in BxPC-3 (**A**–**C**) and DU145 cells (**D**–**F**). Data were normalized to the maximum total uptake and are presented as the average of three dishes (*n* = 3) with SD. Cells were continuously incubated with the radioconjugates at 37 °C. At selected time points, a set of dishes was removed from the incubator and membrane bound and internalized activity were collected. Data on the uptake and internalization of [^68^Ga]Ga-ZHER3 can be found in [27].

To study the internalization of the new radioconjugates, HER3-expresisng BxPC-3 cells were continuously incubated with the compounds for up to 24 h. The total cell uptake and internalized activity of all radioconjugates continuously increased with time (Figure 2A–F). For [^89^Zr]Zr-DFO-seribantumab and [^89^Zr]Zr-DFO-seribantumab-F(ab’)_2_, 31–35% of cell-associated activity was internalized after 24 h of continuous incubation in both cell lines. The internalized fraction of [^68^Ga]Ga-DFO-seribantumab-F(ab’)_2_ was 18 ± 4% and 10 ± 2% of cell associated activity in BxPC-3 and DU145 cells after 4 h, respectively. Uptake and internalization of [^68^Ga]Ga-Z_HER3_ was studied earlier [27].

### 3.5. In Vivo Biodistribution and Specificity

**Figure 3:**
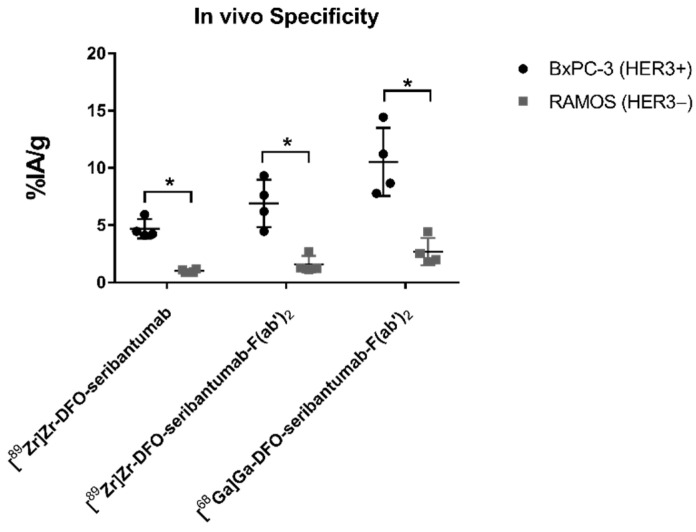
In vivo specificity of [^89^Zr]Zr-DFO-seribantumab, [^89^Zr]Zr-DFO-seribantumab-F(ab’)_2_, and [^68^Ga]Ga-DFO-seribantumab-F(ab’)_2_. Groups of four mice (*n* = 4) bearing either HER3-expressing BxPC-3 xenografts or HER3-negative RAMOS xenografts were injected with 27 µg of ^68^Ga/^89^Zr-labeled DFO-seribantumab-F(ab’) or 35 µg [^89^Zr]Zr-DFO-seribantumab and sacrificed 3 h and 96 h pi, respectively. ^*^ Statistically significant (*p* < 0.05) difference in tumor uptake.

**Figure 4:**
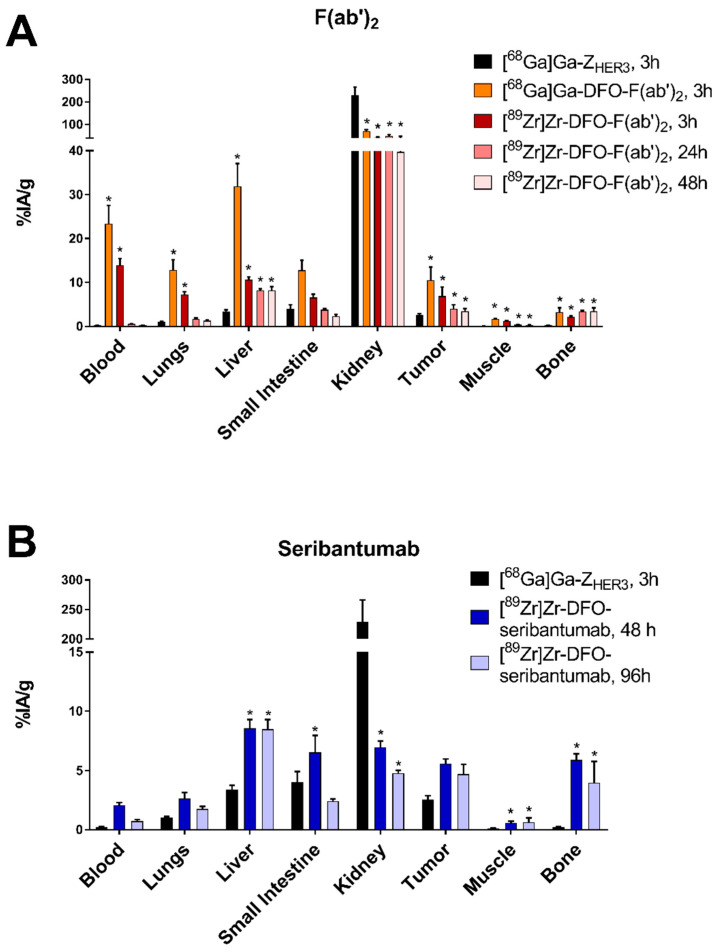
Biodistribution of (**A**) [^68^Ga]Ga-DFO-seribantumab-F(ab’)_2_, [^89^Zr]Zr-DFO-seribantumab-F(ab’)_2_, and (**B**) [^89^Zr]Zr-DFO-seribantumab in balb/c nu/nu mice with HER3-expressing BxPC-3 xenografts. Biodistributions of [^68^Ga]Ga-Z_HER3_ 3 h pi were added to the respective graphs for comparison. Data are presented as the average of *n* = 4–6 animals with SD. * Indicates a statistically significant difference (*p* < 0.05) with [^68^Ga]Ga-Z_HER3_. Numerical data as well as detailed information on statistically significant differences between groups can be found in Supplementary Materials, Tables S1 and S2.

**Figure 5:**
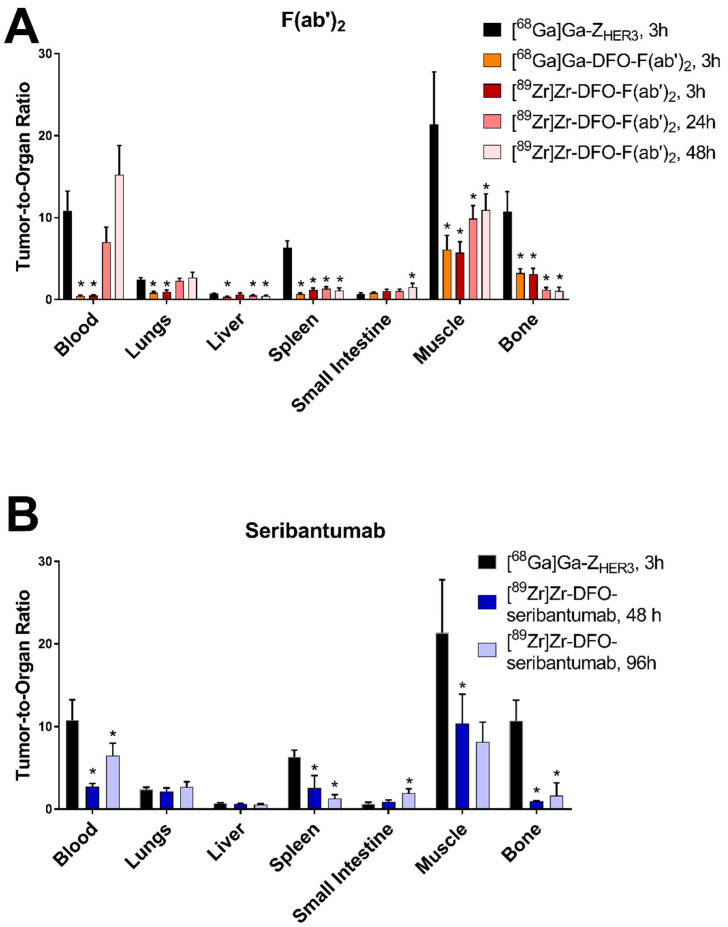
Tumor-to-organ ratios of (**A**) [^68^Ga]Ga-DFO-seribantumab-F(ab’)_2_, [^89^Zr]Zr-DFO-seribantumab-F(ab’)_2_, and (**B**) [^89^Zr]Zr-DFO-seribantumab in balb/c nu/nu mice with HER3-expressing BxPC-3 xenografts. Tumor-to-organ ratios of [^68^Ga]Ga-Z_HER3_ 3 h pi were added to the respective graphs for comparison. Data are presented as the average of *n* = 4–6 animals with SD. * Indicates a statistically significant difference (*p* < 0.05) with [^68^Ga]Ga-Z_HER3_. Numerical data as well as detailed information on statistically significant differences between groups can be found in Supplementary Materials, Tables S1 and S2.

In vivo specificity and biodistribution of the conjugates were studied in Balb/c nu/nu mice with HER3-positive and HER3-negative RAMOS xenografts. Time points were 3 h, 24 h, and 48 h pi for [^89^Zr]Zr-DFO-seribantumab-F(ab’)_2_ and 48 h and 96 h pi for [^89^Zr]Zr-DFO-seribantumab. The measurement time point for [^68^Ga]Ga-DFO-seribantumab-F(ab’)_2_ and [^68^Ga]Ga-Z_HER3_ was 3 h pi. The results from these experiments are displayed in Figure 3, Figure 4 and Figure 5, and the numerical biodistribution data are available in Supplementary Materials, Tables S1 and S2.

Regarding the in vivo specificity test, the uptake of [^89^Zr]Zr-DFO-seribantumab, [^89^Zr]Zr-DFO-seribantumab-F(ab’)_2_, and [^68^Ga]Ga-DFO-seribantumab-F(ab’)_2_ in HER3-negative RAMOS xenografts was significantly lower compared with the uptake in BxPC-3 xenografts (Figure 3), demonstrating HER3-specific binding of the radioconjugates in vivo. Binding specificity of [^68^Ga]Ga-ZHER3 towards HER3 in vivo was confirmed previously [27].

Among all the tested tracers and time points, the uptake in BxPC-3 xenografts was the highest for [^68^Ga]Ga-DFO-seribantumab-F(ab’)_2_ (11 ± 3%ID/g) and [^89^Zr]Zr-DFO-seribantumab-F(ab’)_2_ 3 h pi (7 ± 2%ID/g) (Figure 4). However, the uptake of [^89^Zr]Zr-DFO-seribantumab-F(ab’)_2_ significantly decreased from 3 h to 48 h pi. There was no significant difference in tumor uptake of [^89^Zr]Zr-DFO-seribantumab-F(ab’)_2_ 48 h pi and [^89^Zr]Zr-DFO-seribantumab 48 h and 96 h pi. Tumor uptake of [^68^Ga]Ga-Z_HER3_ was 1.5–4.2-fold lower than the uptake of the antibody-based tracers.

[^68^Ga]Ga-Z_HER3_ showed the fastest clearance from blood circulation. The activity concentration of the affibody in blood 3 h pi was more than 100-fold lower compared with [^68^Ga]Ga-DFO-seribantumab-F(ab’)_2_, 69-fold lower compared with [^89^Zr]Zr-DFO-seribantumab-F(ab’)_2_ 3 h pi, and 10-fold lower than [^89^Zr]Zr-DFO-seribantumab 48 h pi. Concentration of [^68^Ga]Ga-DFO-seribantumab-F(ab’)_2_ in blood 3 h pi was significantly higher than the activity concentration of [^89^Zr]Zr-DFO-seribantumab-F(ab’)_2_ in blood at this time point. Blood concentration of [^89^Zr]Zr-DFO-seribantumab-F(ab’)_2_ further decreased with time, reaching 0.23 ± 0.04%ID/g 48 h pi. This was almost 10-fold lower than the blood concentration of [^89^Zr]Zr-DFO-seribantumab at the same time point. The concentration of [^89^Zr]Zr-DFO-seribantumab in blood decreased three-fold from 48 to 96 h pi.

For [^89^Zr]Zr-DFO-seribantumab, the highest uptake in normal organs at both time points was observed in liver (without significant difference in liver between the time points). The elevated uptake in the stomach, small intestine and GI tract significantly decreased from 48 h to 96 h pi.

At three hours pi, the highest uptake of both DFO-seribantumab-F(ab’)_2_-based tracers was observed in kidneys, blood, liver, lungs, small intestine, and spleen. The uptake of [^68^Ga]Ga-DFO-seribantumab-F(ab’)_2_ in all normal organs was significantly higher than the uptake of [^89^Zr]Zr-DFO-seribantumab-F(ab’)_2_. In most normal organs, the uptake of [^89^Zr]Zr-DFO-seribantumab-F(ab’)_2_ significantly decreased from 3 h to 48 h pi. In liver, small intestine, and kidney, no significant change in uptake was observed after the 24 h time point.

Normal organ uptake (except kidneys) of [^68^Ga]Ga-Z_HER3_ was generally lower compared with the uptake of the ^89^Zr-labeled radioconjugates and [^68^Ga]Ga-DFO-seribantumab-F(ab’)_2_. Most remarkably, the liver, spleen, and bone uptake of [^68^Ga]Ga-Z_HER3_ was significantly lower at all time points time points compared with all other conjugates. The renal uptake for [^68^Ga]Ga-Z_HER3_ was significantly higher than for [^89^Zr]Zr-DFO-seribantumab and [^68^Ga]Ga/[^89^Zr]Zr-DFO-seribantumab-F(ab’)_2_ due to the rapid renal clearance of affibody molecules. The renal uptake of [^68^Ga]Ga-DFO-seribantumab-F(ab’)_2_ and [^89^Zr]Zr-DFO-seribantumab-F(ab’)_2_ were significantly higher compared with the renal uptake of [^89^Zr]Zr-DFO-seribantumab at all time points.

[^68^Ga]Ga-Z_HER3_ had a significantly higher tumor-to-blood ratio than the other radioconjugates at all time points, with the exception of [^89^Zr]Zr-DFO-seribantumab-F(ab’)_2_ 24 and 48 h pi (Figure 5). The tumor-to-blood ratios of [^89^Zr]Zr-DFO-seribantumab-F(ab’)_2_ significantly increased with time. There was a noticeable, however non-significant, increase in the tumor-to-blood ratio for [^89^Zr]Zr-DFO-seribantumab from 48 to 96 h pi. The highest tumor-to-blood ratios for [^89^Zr]Zr-DFO-seribantumab-F(ab’)_2_ and [^89^Zr]Zr-DFO-seribantumab were observed 48 h pi (16 ± 4) and 96 h pi (6 ± 2), respectively. The tumor-to-blood ratio for [^89^Zr]Zr-DFO-seribantumab-F(ab’)_2_ 48 h pi was significantly higher than the tumor-to-blood ratio of [^89^Zr]Zr-DFO-seribantumab at both time points. There was no significant difference between the tumor-to-organ ratios for [^68^Ga]Ga-DFO-seribantumab-F(ab’)_2_ and [^89^Zr]Zr-DFO-seribantumab-F(ab’)_2_, except in the tumor-to-liver ratio, which was almost two-fold lower for [^68^Ga]Ga-DFO-seribantumab-F(ab’)_2_. The other tumor-to-non-tumor ratios for [^68^Ga]Ga-Z_HER3_ 3 h pi were equal or higher than the ratios for [^68^Ga]Ga-DFO-seribantumab-F(ab’)_2_ 3 h pi, [^89^Zr]Zr-DFO-seribantumab-F(ab’)_2_ 48 h pi, and [^89^Zr]Zr-DFO-seribantumab 96 h pi in most organs (except tumor-to-kidney ratios). This included a significantly higher tumor-to-liver ratio for [^68^Ga]Ga-Z_HER3_ than [^89^Zr]Zr-DFO-seribantumab-F(ab’)_2_ 48 h pi, and significantly higher tumor-to-muscle and tumor-to-bone ratios than both [^89^Zr]Zr-DFO-seribantumab-F(ab’)_2_ 48 h pi and [^89^Zr]Zr-DFO-seribantumab 96 h pi.

### 3.6. Ex Vivo Autoradiography of BxPC-3 Xenografts

**Figure 6:**
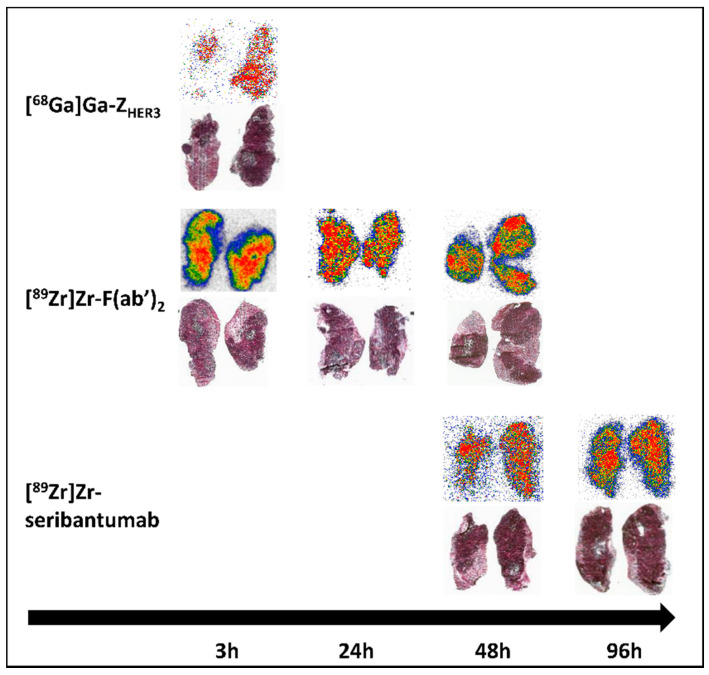
Autoradiography and H&E staining of BxPC-3 xenograft sections. Balb/c nu/nu mice were injected with [^89^Zr]Zr-DFO-seribantumab-F(ab’)_2_, [^89^Zr]Zr-DFO-seribantumab, or [^68^Ga]Ga-Z_HER3_ (equimolar amounts). Mice were sacrificed, and xenografts were excised at matching time points to biodistribution experiments and PET imaging.

Ex vivo autoradiography showed an even distribution of activity in the tumors for all radioconjugates at all time points (Figure 6). The activity accumulation that matched with the localization of tumor cells was higher in tumor tissue than in connective tissue (Figure 6).

### 3.7. nanoPET/CT Imaging

**Figure 7:**
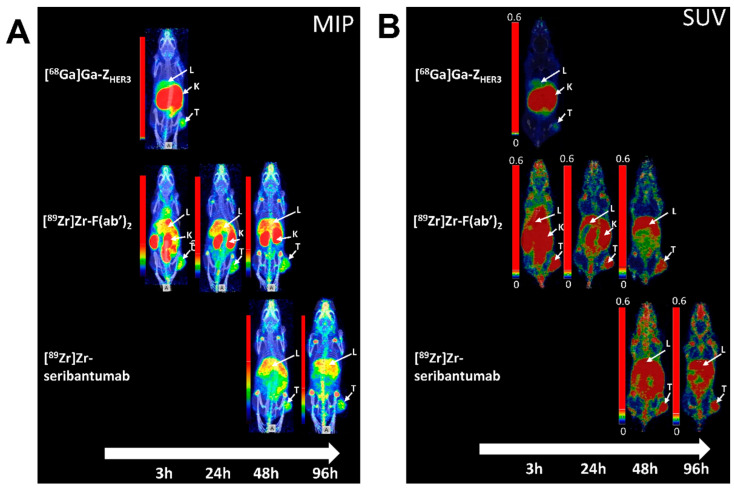
nanoPET/CT images: (**A**) MIP images; (**B**) SUV images of coronal slices. For imaging mice were injected with 1.18 MBq (27 µg) [^89^Zr]Zr-DFO-seribantumab-F(ab’)_2_, 1.38 MBq (35 µg) [^89^Zr]Zr-DFO-seribantumab, or 7.05 MBq (2 µg) [^68^Ga]Ga-Z_HER3_. White arrows indicate tumor (T), kidneys (K), and liver (L).

nanoPET images of [^89^Zr]Zr-DFO-seribantumab-F(ab’)_2_, [^89^Zr]Zr-DFO-seribantumab, and [^68^Ga]Ga-Z_HER3_ were acquired at the respective time points selected for biodistribution and are displayed in Figure 7 and Figure S3. No images were acquired for [^68^Ga]Ga-DFO-seribantumab-F(ab’)_2_ due to the unfavorable biodistribution.

[^89^Zr]Zr-DFO-seribantumab-F(ab’)_2_, [^89^Zr]Zr-DFO-seribantumab, and [^68^Ga]Ga-Z_HER3_ were able to visualize the HER3-expressing BxPC-3 xenografts. Images were in good agreement with the biodistribution results (Tables S1 and S2). High background signal from blood was observed for [^89^Zr]Zr-DFO-seribantumab-F(ab’)_2_ and [^89^Zr]Zr-DFO-seribantumab at early time points, Additionally, high activity uptake for [^89^Zr]Zr-DFO-seribantumab-F(ab’)_2_ and [^89^Zr]Zr-DFO-seribantumab was visible in the liver and GI tract as well as uptake in the bones and joints (Figure 7 and Figure S3).

## 4. Discussion

Upregulation of HER3 is known to be a possible mediator for therapy resistance in cancer [3]. Dynamic changes in HER3 expression and inter-tumor heterogeneity warrant the development of tools that can repeatedly and non-invasively assess the status of HER3 expression in the primary tumor and metastases. PET imaging could provide a repeatable, non-invasive whole-body assessment of HER3 expression to select patients for HER3-targeted therapy. Because of the challenges associated with HER3 as a molecular imaging target (low overexpression and substantial expression in normal tissue), potential tracers need to be carefully designed. It is important that the imaging agent binds the target with high affinity and clears efficiently from the blood and other non-targeted tissues. Several different types of targeting molecules have been explored for radionuclide imaging of HER3 expression [8]. The limited success of antibody-based tracers for PET imaging of HER3 expression suggests that the modification of preexisting therapeutic antibodies might not be sufficient for imaging of a challenging targets such as HER3. Here, we characterized the new HER3-targeting radiotracers [^89^Zr]Zr-DFO-seribantumab and [^68^Ga]Ga/[^89^Zr]Zr-DFO-seribantumab-F(ab’)_2_ in vitro and compared their PET imaging properties at their respective favorable time points with the ^68^Ga-labeled affibody molecule [^68^Ga]Ga-Z_HER3_ in a preclinical mouse model.

A DFO chelator was coupled to seribantumab and seribantumab-F(ab’)_2_ to allow labeling of these proteins either with ^68^Ga or ^89^Zr. The exact ratio of chelate to protein was not determined in this study. However, in a similar published protocol with the same methodology using a DFO:mAb molar ratio of 3:1, conjugation typically resulted in 0.3–0.9 coupled DFO moieties per antibody [31]. Such modification should not dramatically influence the biological properties of the proteins, which was corroborated by our results. Labeling of DFO-seribantumab resulted in quantitative yields. Radiochemical yields for labeling of DFO-seribantumab-F(ab’)_2_ with either ^68^Ga or ^89^Zr could potentially be improved by an increase in temperature, but due to the heat sensitivity of antibodies and antibody fragment labeling, temperature would be limited to physiological temperature. Since purification with size-exclusion columns provided sufficient purity of the product no optimization of the labeling conditions was attempted. The new radioconjugates bound specifically to HER3 in vitro and in vivo and enabled visualization of HER3 expression using nanoPET. Binding affinities of seribantumab and seribantumab-F(ab’)_2_ towards HER3 measured by SPR were lower compared with the affinity values published for radiolabeled Z_HER3_ [29,30]. SPR-analysis furthermore showed cross-reactivity of seribantumab and seribantumab-F(ab’)_2_ to mErbB3 providing improved value to the preclinical model and enabled excellent comparability of the new tracers with [^68^Ga]Ga-Z_HER3_ (with known affinity to mErbB3 [34]).

To our knowledge, no ^89^Zr-labeled version of seribantumab has yet been reported for imaging of HER3 expression. The general pattern of biodistribution, tumor uptake, tumor-to-blood ratio, and hepatic uptake of [^89^Zr]Zr-DFO-seribantumab appear to be comparable to other reported HER3-targeting ^89^Zr-labeled antibodies [17,35,36]. The uptake of [^89^Zr]Zr-DFO-seribantumab was the highest in the liver, due to the known elimination of antibodies via the hepatobiliary pathway. As expected, imaging contrast improved from 48 h to 96 h pi, mainly due to the clearance from blood. This is in agreement with the general expectation for antibody-based tracers and studies of other ^89^Zr-labeled HER3-targeting antibodies that reported the most favorable imaging time points to be between 4 and 7 days pi [11,15,35]. A detailed comparison of the biodistribution of [^89^Zr]Zr-DFO-seribantumab with other HER3-targeting antibody-based tracers is difficult because of differences in the xenograft models and dosing. In our study, the injected dose of [^89^Zr]Zr-DFO-seribantumab was 35 µg, corresponding to 1.75 mg/kg which is close to the ID_50_ of 2 mg/kg determined by Bensch et al. during clinical studies with [^89^Zr]Zr-lumretuzumab [11]. In a study by Alsaid and colleagues, the uptake of [^89^Zr]Zr-GSK2849330 in CHL-1 xenografts was approximately 3.5%ID/g and the tumor-to-blood-ratio was approximately 3.5 48 h pi with an injected dose of 0.5 mg/kg [35]. A further increase in the dose to 1–3 mg/kg also increased the tumor uptake to 8–10%ID/g and tumor-to-blood ratio 7–8 144 h pi.

In the present study, we also investigated a newly produced seribantumab-derived F(ab’)_2_-fragment labeled with ^68^Ga and ^89^Zr. ^89^Zr could be considered an uncontroversial choice for labeling of F(ab’)_2_-fragments. However, antibody fragment-based tracers are known to have faster blood clearance than full-length antibodies and, as a result, could provide the opportunity for imaging at earlier time points. Thus, we also wanted to explore the possibility of a radiolabel with a shorter half-life such as ^68^Ga.

A ^68^Ga-labled variant of a trastuzumab-derived DOTA-conjugated F(ab’)_2_ has been reported for preclinical and clinical imaging of HER2-positive breast cancer 3 h pi [37,38]. However, in this study, ^68^Ga proved to be an unsuitable choice for imaging of HER3 expression with DFO-seribantumab-F(ab’)_2_. The biodistribution of the tracer appeared to be affected by insufficient stability of the [^68^Ga]Ga-DFO complex. Trans-chelation of gallium to transferrin in the blood could be the reason for the observed elevated blood concentration. Besides trans-chelation, gallium-colloids are also known to accumulate in the liver and spleen, both of which sites are where we observed high activity uptake in the biodistribution study of [^68^Ga]Ga-DFO-seribantumab-F(ab’)_2_ [39,40]. The insufficient stability of the [^68^Ga]Ga-DFO complex was also corroborated by the dramatic release of ^68^Ga within a few hours of the in vitro serum challenge. This is in agreement with results from trans-chelation experiments by Brandt et al., where in a direct comparison of [^68^Ga]Ga-DFO and [^89^Zr]Zr-DFO labels, the [^68^Ga]Ga-DFO-complex was significantly less stable during DTPA challenge [41]. We could speculate that the differences observed in the stability of the [^68^Ga]Ga-DFO and [^89^Zr]Zr-DFO complexes might be related to the difference of the ^68^Ga and ^89^Zr ions in size, charge, and preferred coordination sphere which results in different stabilities of the complexes formed with DFO [42,43]. Overall, the general use of the [^68^Ga]Ga-DFO-complex might be a contradictory subject, since the [^68^Ga]Ga-DFO complex has shown sufficient stability in other studies [43,44,45,46]. Regardless, in this study, the clearance and increasing tumor-to-non-tumor contrast, particularly tumor-to-blood contrast, of [^89^Zr]Zr-DFO-seribantumab-F(ab’)_2_ with time indicated that 3 h pi was an unsuitable time point for imaging with [^68^Ga]Ga-DFO-seribantumab-F(ab’)_2_. As a result, no PET images were acquired for this tracer, and it is not included in further discussions.

As anticipated, the clearance of [^89^Zr]Zr-DFO-seribantumab-F(ab’)_2_ from blood was faster than the clearance of [^89^Zr]Zr-DFO-seribantumab due to the size. Matching data were reported for [^111^In]In–cetuximab and [^111^In]In-cetuximab-F(ab’)_2_ [47]. The increased renal uptake of [^89^Zr]Zr-DFO-seribantumab-F(ab’)_2_ compared with [^89^Zr]Zr-DFO-seribantumab was also expected, because it is known that F(ab’)_2_-fragments are primarily catabolized by kidneys due to the size and absence of the Fc-region [48,49].

As opposed to [^89^Zr]Zr-DFO-seribantumab, the tumor uptake of [^89^Zr]Zr-DFO-seribantumab-F(ab’)_2_ decreased with time (two-fold from 3 h to 48 h pi). This could possibly be related to the faster clearance of the F(ab’)_2_-fragement, which shifts the equilibrium in the blood to dissociation. Accordingly, it can be speculated that the stable tumor uptake of [^89^Zr]Zr-seribantumab was due to the steady supply of the tracers from the blood pool. Aside from the uptake in the kidney and tumor, the overall uptake of [^89^Zr]Zr-DFO-seribantumab-F(ab’)_2_ and [^89^Zr]Zr-DFO-seribantumab at 48 h and 96 h pi were comparable in showing uptake in organs with expression of mErbB3 due to the cross-reactivity. Interestingly, this was also the case in the liver. Even though some uptake of [^89^Zr]Zr-DFO-seribantumab-F(ab’)_2_ in liver was expected because of the expression of mErbB3, we had anticipated higher hepatic accumulation of [^89^Zr]Zr-DFO-seribantumab (due to the hepatobiliary excretion of full-length antibodies), as it was shown in a comparison of [^111^In]In-cetuximab and [^111^In]In-cetuximab-F(ab’)_2_ in the past [47]. Because of the highest tumor-to-blood ratios, we considered 96 h and 48 h pi to be the best time for PET imaging with [^89^Zr]Zr-DFO-seribantumab and [^89^Zr]Zr-DFO-seribantumab-F(ab’)_2_, respectively. We would, furthermore, consider [^89^Zr]Zr-DFO-seribantumab-F(ab’)_2_ the more suitable imaging probe compared with [^89^Zr]Zr-DFO-seribantumab due to the significantly higher tumor-to-blood contrast. This was also reported for [^64^Cu]Cu-anti-HER3-F(ab’)_2_, a mAb105-derived fragment and the only other HER3 targeting F(ab’)_2_-fragment reported in the literature, even though no full biodistribution data were published [19].

The biodistribution of [^68^Ga]Ga-Z_HER3_ was in good agreement with earlier published data by our group [27,30]. Despite the lower uptake in xenograft, [^68^Ga]Ga-Z_HER3_ provided equal or higher tumor-to-non-tumor ratios and visibly better PET contrast than [^89^Zr]Zr-DFO-seribantumab and [^89^Zr]Zr-DFO-seribantumab-F(ab’)_2_ because of the lower uptake in normal organs. Both observations are most likely linked to overall faster clearance of Z_HER3_. In particular, we considered [^68^Ga]Ga-Z_HER3_ superior to [^89^Zr]Zr-DFO-seribantumab because of the significantly higher tumor-to-blood ratio. In comparison with [^89^Zr]Zr-DFO-seribantumab-F(ab’)_2_ 48 h pi, the tumor-to-blood ratio of [^68^Ga]Ga-Z_HER3_ was similar, but [^68^Ga]Ga-Z_HER3_ provided significantly better contrast in HER3-expressing organs, particularly in liver, which is a common site for metastases in many cancers.

A ^89^Zr-labeled variant of Z_HER3_ has been reported in the literature [25]. Comparing the available data on [^89^Zr]Zr-DFO-Z_HER3_ with our data, [^89^Zr]Zr-DFO-Z_HER3_ had lower tumor-to-blood contrast than the [^68^Ga]Ga-Z_HER3_ variant 3 h pi [27,30]. It should be mentioned, however, that [^89^Zr]Zr-DFO-Z_HER3_ was injected at a lower dose (1 µg) than [^68^Ga]Ga-Z_HER3_ and studied in an MCF-7 xenograft model. Our group has previously reported that next-day imaging of HER3 using a radiocobalt-labeled Z_HER3_ variant can considerably improve the HER3-PET contrast in mice [30]. For [^89^Zr]Zr-DFO-Z_HER3_, the tumor-to-blood ratio improved from 3 h to 24 h pi, but the tumor-to-non-tumor contrast in other tissues remained the same or decreased [25]. This suggests that [^89^Zr]Zr-DFO-Z_HER3_ might not be the optimal configuration for a Z_HER3_-based imaging agent. Still, the PET-image contrast of [^89^Zr]Zr-DFO-Z_HER3_ could be considered favorable to [^89^Zr]Zr-DFO-seribantumab.

In addition to the clear advantage in imaging contrast, using the ^68^Ga-labeled affibody may provide several other advantages for PET imaging of HER3 expression. Release of ^89^Zr from the [^89^Zr]Zr-DFO complex in vivo is a known phenomenon, occurring most likely to the unsaturated coordination sphere of Zr(IV) by DFO [41]. Free ^89^Zr is known to accumulate in the bones and particularly joints [50], which was clearly visualized on the PET images in this study. This is an obvious disadvantage for imaging of bone metastasis, which are also known to express HER3 [2,51]. A longer time between tracer injection and acquisition of the scan, as is the case for antibody-based tracers, even further exacerbates the problem of ^89^Zr accumulation in normal tissues due to the continuous release of the nuclide during that period. Stability of the [^89^Zr]Zr-DFO complex could potentially be improved by increasing the temperature during radiolabeling [46]. This, however, is not a suitable option for the labeling of antibodies and antibody-fragments, because they are not able to withstand heating above physiological temperature. Another possibility to reduce the release of ^89^Zr is the use of the recently developed DFO-derivative DFO* [52]. [^89^Zr]Zr-DFO* has shown improved stability compared with [^89^Zr]Zr-DFO for labeling of trastuzumab, resulting in significant reduction in bone uptake [53]. The faster clearance of [^68^Ga]Ga-Z_HER3_ also reduces the radiation dose to the patients. For example, for the HER3-targeting antibody [^89^Zr]Zr-GSK2849330, the effective radiation dose to patients was reported to be 0.46–0.59 mSv/MBq [15], whereas the effective dose for the HER2-targeting affibody [^68^Ga]Ga-ABY-025 was only 0.030 ± 0.003 mSv/MBq [54]. Furthermore, the faster clearance of [^68^Ga]Ga-Z_HER3_ would enable repeat scanning within a shorter time. Additionally, an image time point shortly after injection could be better suited to detect rapidly occurring HER3 expression changes or image receptor occupancy during HER3-targeted therapy as in [24].

## 5. Conclusions

In conclusion, we have shown that the choice of targeting molecule can have a profound impact on HER3-PET image contrast. While the approach of using pre-existing therapeutic monoclonal antibodies for imaging of HER3 expression might be appealing, the complicated nature of HER3 as a molecular imaging target might necessitate the design of designated probes for molecular imaging in order to provide sufficient imaging contrast. Even though all studied tracers were able to visualize HER3 expression in our preclinical model, the results of this study suggest that smaller imaging agents, such as affibody molecules, are more suitable for PET imaging of HER3 expression than full-length antibody and antibody-fragment-based agents and warrant further translational studies.

## Figures and Tables

**Table 1:** Radiochemical yield, purity, and stability of [^89^Zr]Zr-DFO-seribantumab, [^89^Zr]Zr-DFO-seribantumab-F(ab’)_2_, and [^68^Ga]Ga-DFO-seribantumab-F(ab’)_2_ determined by radio-iTLC. Stability is expressed as % protein-associated activity. Because of the almost quantitative radiochemical yield, [^89^Zr]Zr-DFO-seribantumab was used without further purification. Data on the in vitro stability of [^68^Ga]Ga-Z_HER3_ was published earlier and can be found in reference [27].

Radioconjugate	Radiochemical Yield (%) (*n* = 3)	Purity (%)	Stability PBS (%)			Stability Serum 37 °C (%)		
			1 h	3 h	24 h	1 h	3 h	24 h
[^89^Zr]Zr-DFO-seribantumab	99.7 ± 0.5	-	100 ± 0	100.0 ± 0.1	100 ± 0	97.0 ± 0.7	92 ± 3	76 ± 4
[^89^Zr]Zr-DFO-seribantumab-F(ab’)_2_	80 ± 4	97 ± 1	97.1 ± 0.6	96.4 ± 0.8	92.4 ± 1.0	94.1 ± 0.8	93 ± 2	77 ± 6
[^68^Ga]Ga-DFO-seribantumab-F(ab’)_2_	88 ± 2	100 ± 0	100 ± 0	100 ± 0	-	15 ± 2	17 ± 3	-

## Data Availability

Data is contained within the article or Supplementary Material.

## Supplementary Materials

The following are available online at https://www.mdpi.com/article/10.3390/cancers13194791/s1, Figure S1: SDS-PAGE showing molecular weight marker (lane 1), intact seribantumab (lane 2), seribantumab-F(ab’)_2_ after pepsin digestion (lane 3), and successful removal of intact seribantumab and Fc fragments by SEC purification (lane 4); Figure S2: Representative SPR sensorgrams showing binding kinetics against immobilized (A) human HER3 and (B) murine ErbB3 for seribantumab and seribantumab-F(ab’)_2_; Figure S3: nanoPET/CT images, sagittal view; Table S1: Biodistribution of [^68^Ga]Ga-DFO-seribantumab-F(ab’)_2_, [^89^Zr]Zr-DFO-seribantumab-F(ab’)_2_, [^89^Zr]Zr-DFO-seribantumab and [^68^Ga]Ga-Z_HER3_ in balb/c nu/nu mice with HER3-expressing BxPC-3 xenografts presented as %ID/g; Table S2: Tumor-to-organ ratios for [^68^Ga]Ga-DFO-seribantumab-F(ab’)_2_, [^89^Zr]Zr-DFO-seribantumab-F(ab’)_2_, [^89^Zr]Zr-DFO-seribantumab, and [^68^Ga]Ga-Z_HER3_ in balb/c nu/nu mice with HER3-expressing BxPC-3 xenografts.
