# Supplementary material for: HER3 PET Imaging: 68Ga-Labeled Affibody Molecules Provide Superior HER3 Contrast to 89Zr-Labeled Antibody and Antibody-Fragment-Based Tracers
Source: Cancers (Basel). 2021 Sep 24;13(19):4791. doi: 10.3390/cancers13194791 (PMC8508546; doi:10.3390/cancers13194791)
Supplement: Supplementary file 1 [file cancers-13-04791-s001.zip › cancers-1369900-supplementary.pdf]

Supplementary Material

HER3 PET-imaging: ^{68}Ga-labeled affibody molecules provide superior HER3 contrast to ^{89}Zr-labeled antibody and antibody-fragment based tracers

Results

Characterization

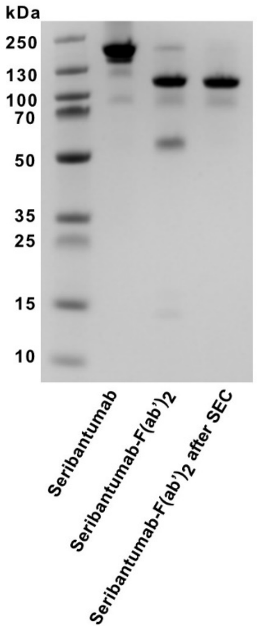

Figure S1: SDS-PAGE showing molecular weight marker (lane 1), intact seribantumab (lane 2), seribantumab-F(ab')$_2$ after pepsin digestion (lane 3), and successful removal of intact seribantumab and Fc fragments by SEC purification (lane 4).

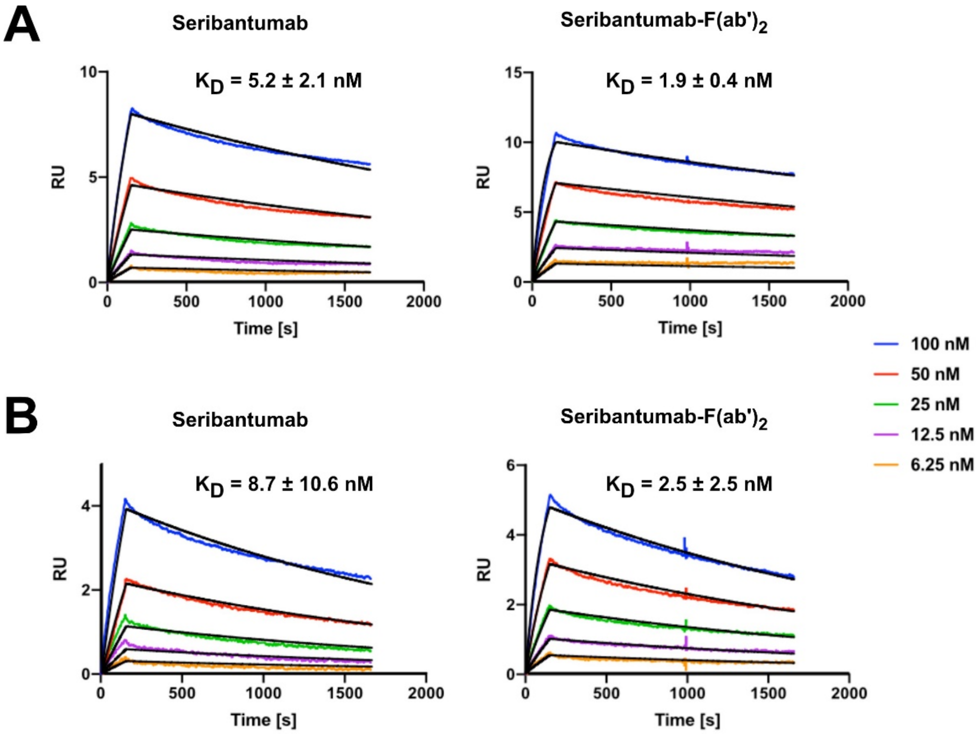

Figure S2: Representative SPR sensorgrams showing binding kinetics against immobilized (A) human HER3 and (B) murine ErbB3 for seribantumab and seribantumab-F(ab′)$_2$. The experimental data (colored curves) were fitted using a Langmuir 1:1 model (black curves) to approximate kinetic constants. The K_D-values (± SD) are presented as the average obtained from two surfaces with different immobilization levels.

In vivo studies

Table S1: Biodistribution of [^{68}Ga]Ga-DFO-seribantumab-F(ab′)$_2$, [^{89}Zr]Zr-DFO-seribantumab-F(ab′)$_2$, [^{89}Zr]Zr-DFO-seribantumab and [^{68}Ga]Ga-Z$_{HER3}$ in balb/c nu/nu mice with HER3-expressing BxPC-3 xenografts presented as %ID/g. Data is presented as average of n=4-6 mice with SD. Data for GI-tract and body is presented as %ID. Significant difference ($p<0.05$) between groups is indicated by [a] vs [^{68}Ga]Ga-DFO-seribantumab-F(ab′)$_2$ [b]vs. [^{89}Zr]Zr-DFO-seribantumab-F(ab′)$_2$ 3 h pi, [c] vs. [^{89}Zr]Zr-DFO-seribantumab-F(ab′)$_2$ 24 h pi, [d] vs. [^{89}Zr]Zr-DFO-seribantumab-F(ab′)$_2$ 48 h pi, [e] vs. [^{89}Zr]Zr-DFO-seribantumab 48 h pi, [f]vs. [^{89}Zr]Zr-DFO-seribantumab 96 h pi, [*]vs. [^{68}Ga]Ga-Z$_{HER3}$. Statistical significance was done by two-tailed unpaired t-test, except for comparison of data from [^{68}Ga]Ga-DFO-seribantumab-F(ab′)$_2$ and [^{89}Zr]Zr-DFO-seribantumab-F(ab′)$_2$ 3 h pi when a two-tailed paired t-test was used, due to a dual isotope approach.

**Organ**	**[^{68}Ga]Ga-DFO-seribantumab-F(ab′)$_2$**	**[^{89}Zr]Zr-DFO-seribantumab-F(ab′)$_2$**			**[^{89}Zr]Zr-DFO-seribantumab**		**[^{68}Ga]Ga-Z$_{HER3}$**
	**3 h pi**	**3h pi**	**24 h pi**	**48 h pi**	**48 h pi**	**96 h pi**	**3 h pi**
Blood	23 ± 4[b,c,d,e,f,*]	14 ± 2[a,c,d,e,f,*]	0.58 ± 0.04[a,b]	0.23 ± 0.04[a,b]	2.1 ± 0.2[a,b]	0.7 ± 0.1[a,b]	0.2 ± 0.07[a,b]
Salivary	4 ± 0.9[b,c,d,f,*]	2.6 ± 0.3[a,c,d,*]	1.9 ± 0.2[a,b,e,f]	1.4 ± 0.2[a,b,e,f]	3.6 ± 0.5[c,*]	3.1 ± 0.3[a,c,*]	1.3 ± 0.1[a,b,e,f]
Lungs	13 ± 2[b,c,d,e,f,*]	7.3 ± 0.6[a,c,d,e,f,*]	1.7 ± 0.2[a,b]	1.3 ± 0.1[a,b]	2.7 ± 0.5[a,b]	1.8 ± 0.2[a,b]	1.05 ± 0.08[a,b]
Liver	32 ± 5[b,c,d,e,f,*]	10.6 ± 0.6[*]	8.2 ± 0.3[a,*]	8.2 ± 0.9[a,*]	8.6 ± 0.7[a,*]	8.5 ± 0.8[a,*]	3.4 ± 0.4[a,b,c,d,e,f]
Stomach	3.3 ± 0.5[b,c,d,e,f,*]	2.6 ± 0.3[a,c,d]	1.5 ± 0.2[a,b]	1.1 ± 0.2[a,b,f]	2.1 ± 0.4[a,f,*]	1.3 ± 0.1[a,d,e]	1.4 ± 0.2[a,b,e]
Spleen	16 ± 3[b,c,d,e,f,*]	5.9 ± 0.6[a,c,d,*]	3.0 ± 0.4[a,b,*]	3.1 ± 0.3[a,b,*]	3 ± 1[a,b,*]	3.7 ± 0.6[a,*]	0.39 ± 0.0[a,b,c,d,e,f]
Small Intestine	13 ± 2[b,c,d,e,f,*]	6.6 ± 0.7[a]	3.8 ± 0.2[a,b,d]	2.3 ± 0.4[a,b,c,e]	7 ± 1[a,d,f,*]	2.4 ± 0.2[a,b,e]	4.0 ± 0.9[a,b,e]
Kidney	70 ± 7[e,f,*]	41 ± 3[e,f,*]	48 ± 7[e,f,*]	40 ± 8[e,f,*]	7.0 ± 0.5[a,b,c,d,*]	4.8 ± 0.2[a,b,c,d,*]	230 ± 37[a,b,c,d,e,f]
Tumor	11 ± 3[b,c,d,e,f,*]	7 ± 2[a,d,*]	4.0 ± 0.9[a]	3.5 ± 0.6[a,b]	5.6 ± 0.4[a]	4.7 ± 0.8[a]	2.6± 0.3[a,b]
Muscle	1.72 ± 0.10[b,c,d,e,f,*]	1.19 ± 0.10[a,c,d,e,f,*]	0.41 ±0.06[a,b]	0.32 ± 0.03[a,b]	0.6 ± 0.2[a,b,*]	0.7 ± 0.4[a,b,*]	0.12 ± 0.02[a,b,e,f]
Bone	3.3 ± 1.0[e,*]	2.2 ± 2[e,*]	3.4 ± 0.2[e,*]	3.5 ± 0.7[e,*]	5.9 ± 0.5[a,b,c,d,*]	4 ± 2[*]	0.24 ± 0.05[a,b,c,d,e,f]
GI	19 ± 2 [c,d,e,f,*]	17 ± 7[c,d,e,f,*]	7 ± 1[a,b]	3.3 ± 0.3[a,b,e]	11 ± 0.9[a,b,d,f]	4.7 ± 0.6[a,b,e]	6 ± 1[a,b]
Body	38 ± 6[b,c,d,e,f,*]	19 ± 9[a,*]	16 ± 1[a,*]	14 ± 1[a,e]	25 ± 2[a,d]	21 ± 1[a,*]	6.3 ± 0.7[a,b,c,d,f,*]

Table S2: Tumor-to-organ ratios for [^{68}Ga]Ga-DFO-seribantumab-F(ab')$_2$, [^{89}Zr]Zr-DFO-seribantumab-F(ab')$_2$, [^{89}Zr]Zr-DFO-seribantumab and [^{68}Ga]Ga-Z$_{HER3}$ in balb/c nu/nu mice with HER3-expressing BxPC-3 xenografts. Data is presented as average of n=4-6 mice with SD. Significant difference (p<0.05)) between groups is indicated by ^{a} vs [^{68}Ga]Ga-DFO-seribantumab-F(ab')$_2$ ^{b}vs. [^{89}Zr]Zr-DFO-seribantumab-F(ab')$_2$ 3 h pi, ^{c}vs. [^{89}Zr]Zr-DFO-seribantumab-F(ab')$_2$ 24 h pi, ^{d} vs. [^{89}Zr]Zr-DFO-seribantumab-F(ab')$_2$ 48 h pi, ^{e}vs. [^{89}Zr]Zr-DFO-seribantumab 48 h pi, ^{f}vs. [^{89}Zr]Zr-DFO-seribantumab 96 h pi, *vs. [^{68}Ga]Ga-Z$_{HER3}$. Statistical significance was done by two-tailed unpaired t-test, except for comparison of data from [^{68}Ga]Ga-DFO-seribantumab-F(ab')$_2$ and [^{89}Zr]Zr-DFO-seribantumab-F(ab')$_2$ 3 h pi when a two-tailed paired t-test was used, due to a dual isotope approach.

Organ	[^{68}Ga]Ga-DFO-seribantumab-F(ab')$_2$	[^{89}Zr]Zr-DFO-seribantumab-F(ab')$_2$			[^{89}Zr]Zr-DFO-seribantumab		[^{68}Ga]Ga-Z$_{HER3}$
	3 h pi	3h pi	24 h pi	48 h pi	48 h pi	96 h pi	3 h pi
Blood	0.45 ± 0.09^{c,d,f,*}	0.5 ± 0.1^{c,d,f,*}	7 ± 2^{a,b,d}	15 ± 4^{a,b,c,e,f}	2.7 ± 0.4^{d,*}	6 ± 2^{a,b,d,*}	11 ± 2^{a,b,e,f}
Salivary	2.6 ± 0.6^{e,f}	2.6 ± 0.5^{e,f}	2.1 ± 0.3	2.4 ± 0.7	1.6 ± 0.2^{a,b}	1.5 ± 0.3^{a,b}	2.0 ± 0.3
Lungs	0.8 ± 0.2^{c,e,d,f,*}	0.9 ± 0.2^{c,d,e,f,*}	2.3 ± 0.3	2.7 ± 0.6	2.2 ± 0.4	2.7 ± 0.6	2.4 ± 0.3
Liver	0.33 ±0.05^{b,e,*}	0.6 ± 0.2	0.5 ± 0.1*	0.4 ± 0.1*	0.66 ± 0.08	0.6 ± 0.1	0.74 ± 0.05^{a,c,d}
Stomach	3 ± 1	2.6 ± 0.6	2.6 ± 0.2	3 ± 1	2.7 ± 0.06	3.6 ± 0.7*	1.9 ± 0.2^{f}
Spleen	0.7 ± 0.1^{e,*}	1.2 ± 0.3*	1.3 ± 0.2*	1.1 ± 0.3*	3 ± 1^{a,*}	1.3 ± 0.4*	6.4 ± 0.8^{a,b,c,d,e,f}
Small Intestine	0.8 ± 0.1^{c,f}	1.0 ± 0.2^{f}	1.1 ± 0.2^{a,f}	1.5 ± 0.5*	0.9 ± 0.2^{f}	2.0 ± 0.5^{a,b,c,e,*}	0.7 ± 0.2^{d}
Kidney	0.1 ± 0.03^{e,f}	0.17 ± 0.04^{e,f}	0.08 ± 0.02^{e,f}	0.09 ± 0.03^{e,f}	0.81 ± 0.07^{a,b,c,d,f,*}	1.0 ± 0.2^{a,b,c,d,e,*}	0.011 ± 0.003^{e,f}
Muscle	6 ± 2*	6 ± 1*	10 ± 2*	11 ± 2*	10 ± 4*	8 ± 2*	21 ± 6^{a,b,c,d,e,f}
Bone	3.2 ± 0.5*	3.1 ± 0.7*	1.2 ± 0.3*	1.1 ± 0.5*	0.95 ±0.09*	2 ± 2*	11 ± 2^{a,b,c,d,e,f}

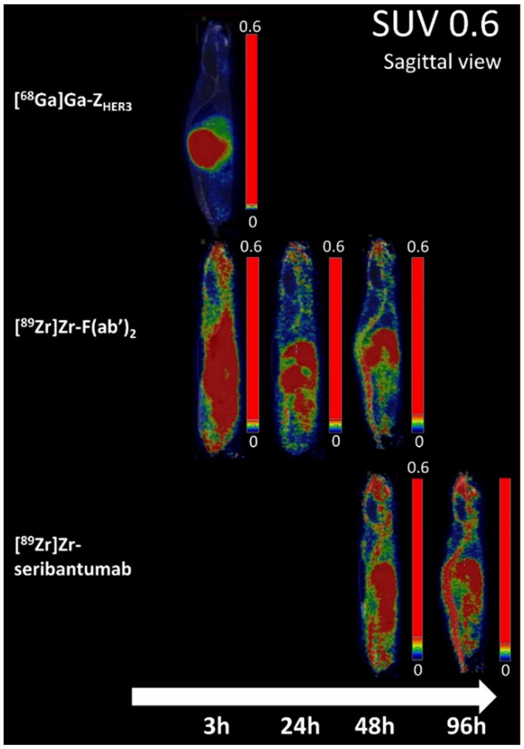

Figure S3: nanoPET/CT images, sagittal view. For imaging mice were injected with 1.18 MBq (27 μg) [^{89}Zr]Zr-DFO-seribantumab-F(ab')$_2$, 1.38 MBq (35 μg) [^{89}Zr]Zr-DFO-seribantumab, or 7.05 MBq (2 μg) [^{68}Ga]Ga-Z$_{HER3}$.
